# Supplementary figures and images for: Comprehensive Transcriptomic Comparison between Porcine CD8− and CD8+ Gamma Delta T Cells Revealed Distinct Immune Phenotype
Source: Animals (Basel). 2021 Jul 22;11(8):2165. doi: 10.3390/ani11082165 (PMC8388496; doi:10.3390/ani11082165)

**Figure S1.**

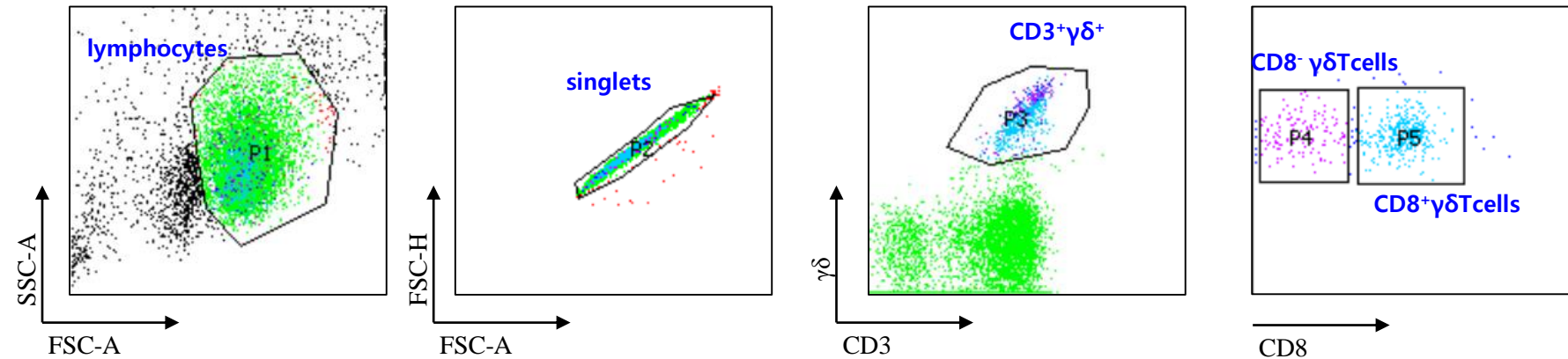

Supplement: Supplementary file 1 [file animals-11-02165-s001.zip › Figure S1.pdf]

Figure S2.

A

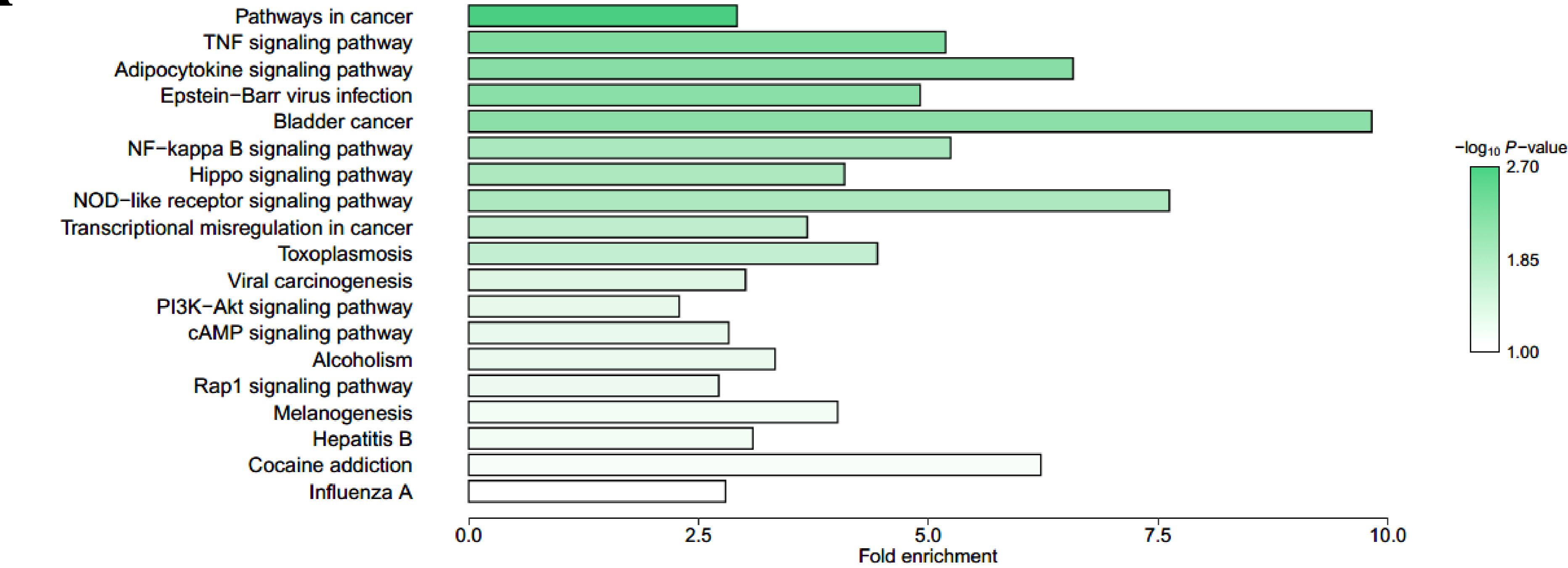

B

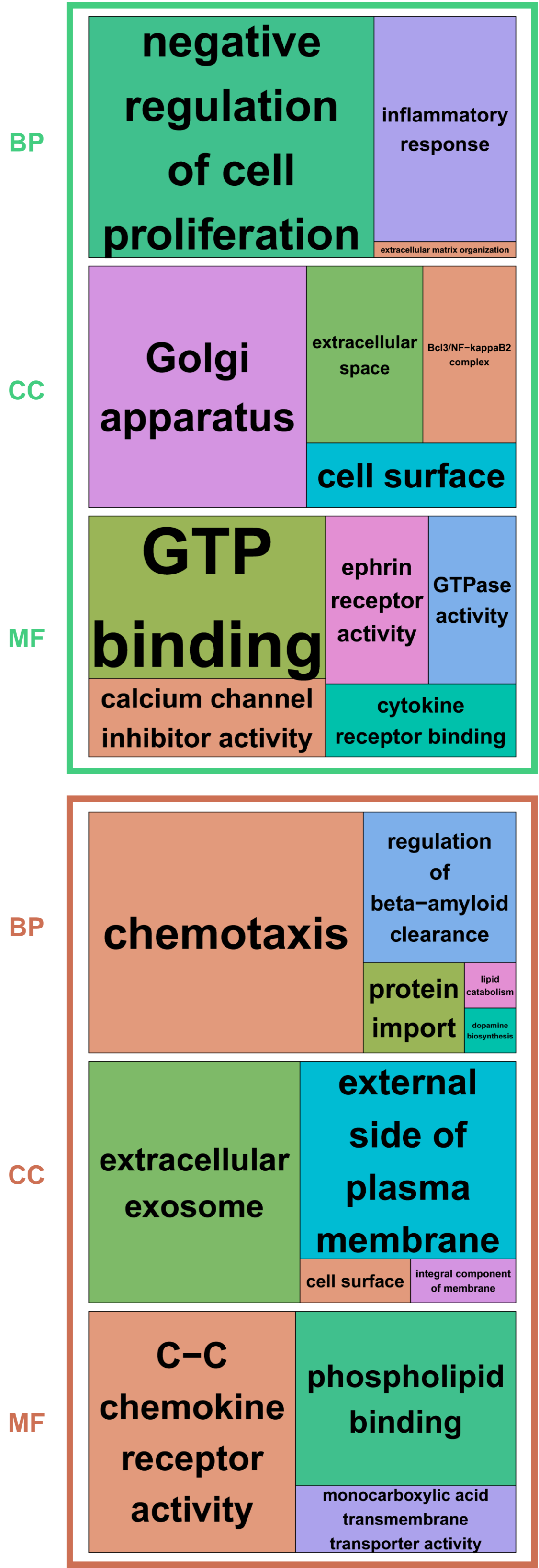

Supplement: Supplementary file 1 [file animals-11-02165-s001.zip › Figure S2.pdf]
